# Use of axillary vascular grafts in prophylactic lymphovenous bypass: technical challenges and lessons learned

**DOI:** 10.3389/fsurg.2026.1821731

**Published:** 2026-05-08

**Authors:** Jorge Flores Garcia, J. Michael Smith, Ramin Rajaii, Emily L. Geisler, Roman J. Skoracki, Min-Jeong Cho

**Affiliations:** 1 Department of Plastic and Reconstructive Surgery, The Ohio State University Wexner Medical Center, Columbus, OH, United States; 2 Division of Plastic Surgery, The University of Texas Medical Branch, Galveston, TX, United States

**Keywords:** breast cancer-related lymphedema, immediate lymphatic reconstruction, lymphatic reconstruction, lymphedema, lymphovenous bypass, microsurgery, prophylactic lymphovenous bypass, vascular graft

## Abstract

**Background:**

Breast cancer–related lymphedema (BCRL) is a common and debilitating complication of axillary lymph node dissection (ALND), affecting approximately 1 in 5 patients. While preventive lymphatic surgeries such as prophylactic lymphovenous bypass (pLVB) performed at the time of ALND have been shown to decrease the risk of developing BCRL, success is frequently limited by inadequate recipient veins within the axilla. In this study, we report our experience with utilizing axillary vascular grafts harvested from within the axillary dissection field to perform pLVB in vein-depleted axillae to prevent the development of BCRL in patients undergoing ALND.

**Methods:**

A retrospective review was performed of breast cancer patients undergoing ALND with planned pLVB at a single institution. Patients in whom axillary vascular grafts were used to enable lymphatic reconstruction were included. Demographic, oncologic, operative, and graft-specific variables were collected. Outcomes included successful lymphatic reconstruction and development of BCRL, assessed using a standardized institutional surveillance protocol incorporating circumferential measurements or perometry.

**Results:**

Thirty-two patients had a mean age of 52.3 years (SD, 12.9) and median BMI of 26.9 kg/m^2^ (IQR, 23.8–28.7), with median follow-up of 15 months (IQR, 11–31). Of the 121 transected lymphatic vessels, interposition grafts were required for 74.8% of reconstructions. A total of 35 grafts (74.3% venous, 25.7% arterial) with median length of 5 cm (IQR, 4–6) were performed with a median of 3 pLVBs (IQR, 2–5) performed per patient. BCRL developed in 6 patients (18.8%) with an average follow-up of 21.3 months (IQR, 11−31); those affected had a higher mean number of lymph nodes removed (22.3 vs. 15.4; *P* < .05), and as a result, more graft-based pLVBs (median 4.5 vs. 2; *P* < .05).

**Conclusion:**

Axillary-based vascular grafting is a versatile strategy that expands reconstructive capacity during pLVB without additional donor-site morbidity. This approach enables completion of lymphatic reconstruction in anatomically challenging, vein-depleted axillae and represents a practical adjunct for immediate lymphatic reconstruction in high-risk breast cancer patients.

## 1 Introduction

Breast cancer-related lymphedema (BCRL) is a chronic, debilitating condition arising from locally and systemically aggressive breast cancer therapy. Approximately one in five breast cancer patients will develop BCRL following oncologic treatment, with axillary node dissection (ALND) being the most substantial risk factor (1–3). In absence of curative treatment, BCRL imposes substantial physical, financial, and psychosocial burdens on affected patients (4). Historically, treatment has focused on management of established disease; however, there has been an increased emphasis placed on preventive strategies (5). The emergence of prophylactic procedures, such as lymphedema microsurgical preventive healing approach (LYMPHA), immediate lymphatic reconstruction (ILR) or prophylactic lymphovenous bypass (pLVB), have demonstrated promise in mitigating the risk of BCRL by preserving lymphatic outflow through anastomosis of transected lymphatics to nearby venous channels at the time of ALND (6–9). Notably, comparative data demonstrates a marked reduction in BCRL incidence among patients undergoing ILR compared with controls at 2-year follow-up (9.2% vs 32%), highlighting the benefits of prophylactic lymphatic reconstruction (10).

Despite clinical promise, supermicrosurgical prophylactic procedures remain technically demanding. A major challenge of the pLVB procedure is the availability of a suitable recipient veins in the axillary region (11). Inadequate vessel length and unfavorable venous characteristics, particularly excessive venous back flow, frequently preclude successful anastomosis creation and represent a leading cause of aborted procedures (12, 13). To address this challenge, Friedman et al. described the use of lower extremity vein grafts to bridge lymphatic–venous gaps, improving pLVB completion rates without increasing operative time (14). However, harvesting grafts from distant donor sites introduces additional surgical site. Herein, we report our institutional experience utilizing venous and arterial grafts harvested entirely within the axillary dissection field to facilitate pLVB in 32 patients, thereby enabling immediate lymphatic reconstruction while avoiding secondary donor sites.

## 2 Materials and methods

### 2.1 Study design

A retrospective review was performed of breast cancer patients undergoing ALND with planned pLVB at The James Cancer Hospital and the Stephanie Spielman Breast Center at The Ohio State University. Approval by the Huron Institutional Review Board at The Ohio State University was obtained prior to study initiation (IRB No. 2024C0098), and the requirement for individual informed consent was waived due to the retrospective nature of the study.

All patients in whom pLVB was performed at the time of ALND using at least one interposition vascular graft to facilitate lymphatic reconstruction were included. Additional inclusion criteria were as follows: patients age 18 years or older, female sex, ALND performed for breast cancer treatment, and postoperative evaluation by lymphedema physiotherapy. Exclusion criteria included preexisting lymphedema or known lymphatic disorders, prior axillary surgery involving either upper extremity, prior ipsilateral or contralateral breast cancer, and lack of postoperative follow-up evaluation.

Patient demographic and clinical variables were obtained through review of the electronic medical record and included age, body mass index (BMI), oncologic treatment history (neoadjuvant and adjuvant chemotherapy and radiation therapy), and duration of follow-up. Oncologic variables collected included the total number of lymph nodes removed and the number of positive lymph nodes identified at ALND. Operative variables collected included the number of lymphatic vessels identified, the number successfully reconstructed, the total number of pLVBs performed per patient, and whether pLVBs were performed with or without interposition grafts. Graft-specific variables included graft type (venous or arterial), graft length, graft donor vessel, recipient vessel, and the use of single vs. serial graft configurations. Lymphatic vessel diameter, anastomotic technique, and intraoperative confirmation of patency using indocyanine green (ICG) imaging were also recorded. Total operative duration was abstracted and reflects all procedures performed in conjunction with ALND and lymphatic reconstruction.

Postoperative outcomes included the development of breast cancer–related lymphedema (BCRL), as determined by clinical documentation and the institutional surveillance protocol. In patients for whom perometry data were unavailable, circumferential measurements were used to assess lymphedema status.

### 2.2 Surgical technique

**Figure 1:**
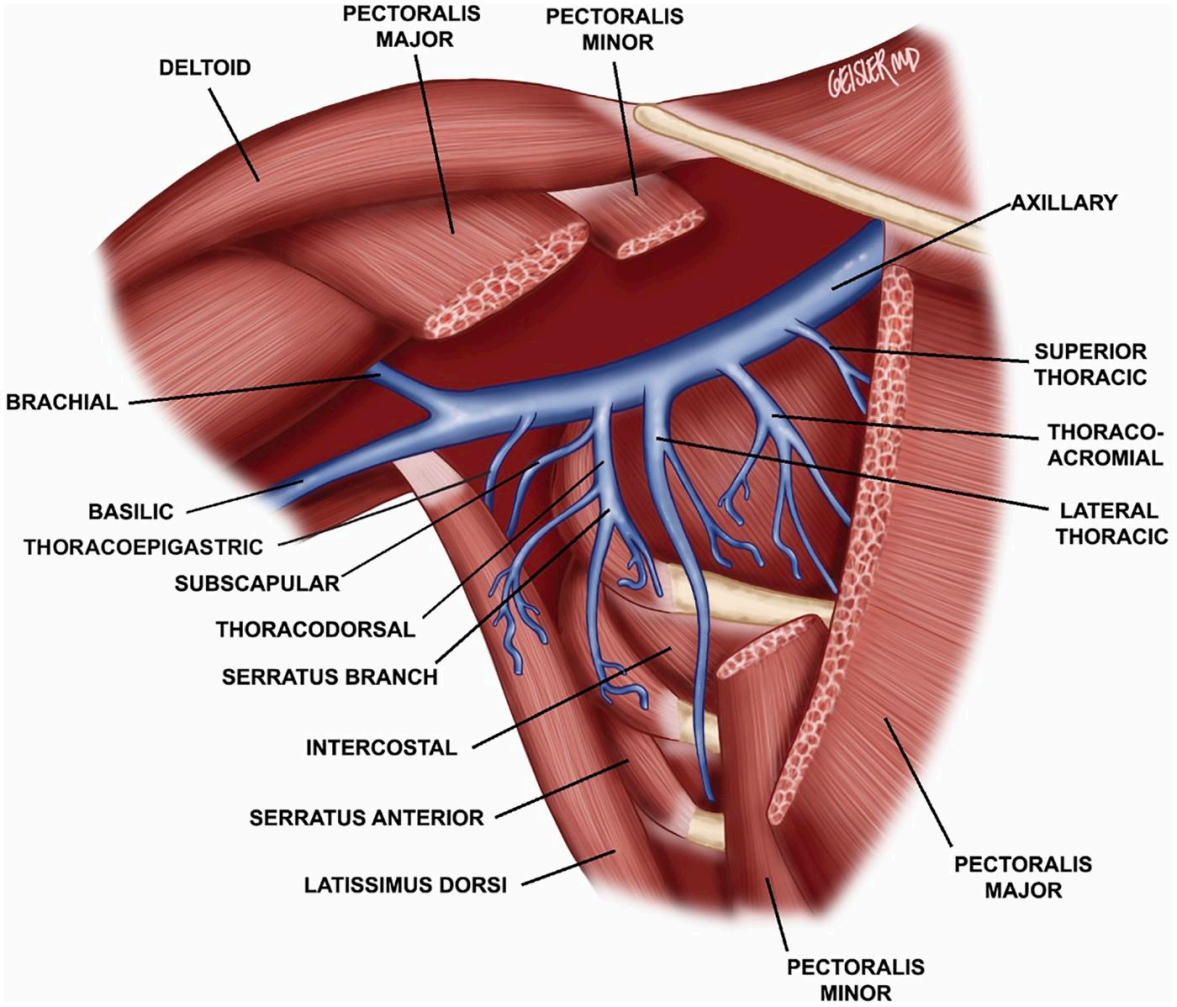
Diagram of axllary venous anatomy including the thoracoepigastric/accessory vein, lateral thoracic vein, serratus vein branches, and axillary vein branches.

**Figure 2:**
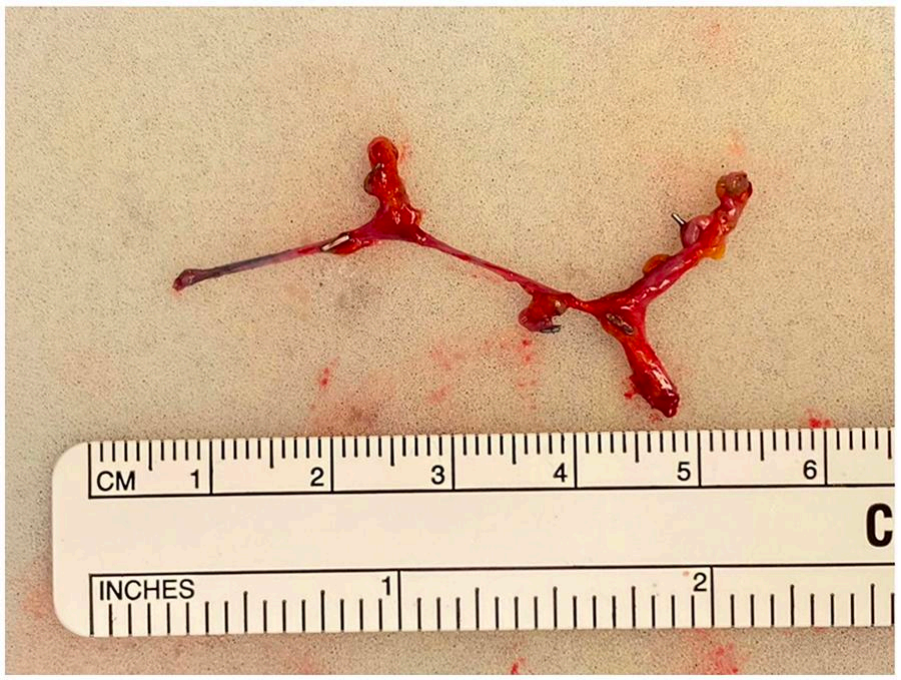
Photograph of harvested axillary vein graft.

**Figure 3:**
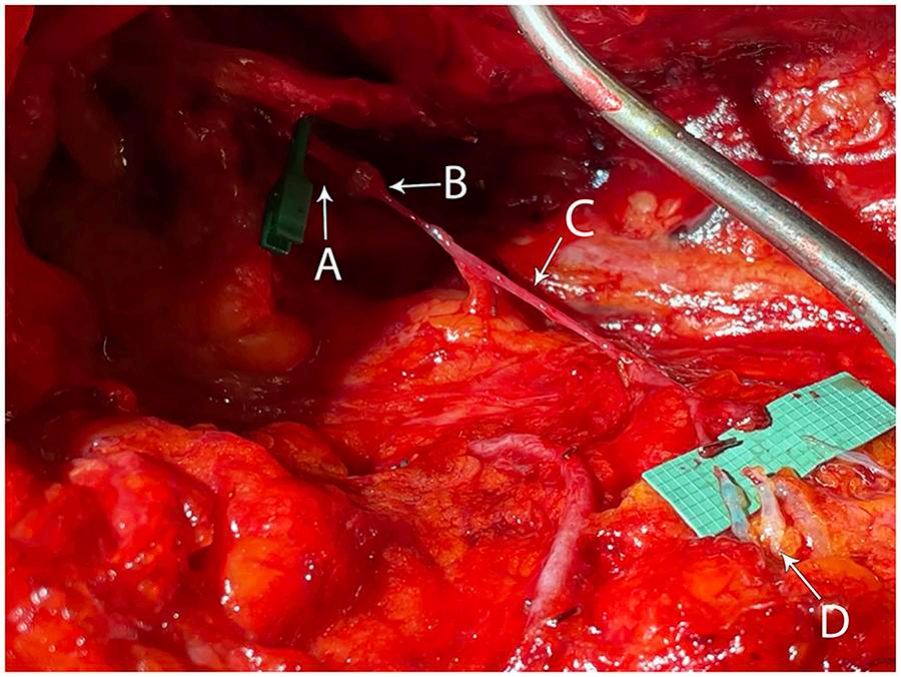
Intraoperative photograph demonstrating interposition of an axillary vein graft prior to lymphatic anastomoses with labeled anatomic structures including axillary vein **(A)**, vascular coupler **(B)**, vein graft **(C)**, and lymphatic channels **(D).**

Prior to the beginning of the ALND, 0.1 cc aliquots of ICG are subdermally injected into the dorsal digital web spaces, the central palm, and the radial and ulnar volar wrist crease. Following completion of the ALND, 0.1cc aliquots of isosulfan blue is injected circumferentially around the upper extremity at the level of the deltoid insertion for further assistance with the identification of transected lymphatic vessels using a near infrared camera. We then inspect the axilla for recipient veins in a systematic manner, starting with an intact tributary of the axillary system (Figure 1) (15). In the absence of such a tributary or if a given side-branch of the axillary vein is too short to reach the cut lymphatic channels without tension, we will harvest vein grafts (Figure 2). Whenever possible, we favor vein grafts that contain a valve to minimize venous backflow. Of note, we try to avoid harvesting the thoracodorsal vein due to its large size and risk of back-bleeding as well as to preserve this potential breast reconstruction salvage option. Grafts are flushed with heparinized saline and interposed between the severed lymphatic channels and recipient veins. The vascular graft is first connected to the recipient vein using hand-sewn end-to-end or end-to-side microsurgical anastomosis or with a venous coupler in select cases in order to bridge the gap to an acceptable downstream recipient vessel. The lymphatic channels are then subsequently connected to the end or side branches of the graft using either end-to-end or intussusception microsurgical techniques (Figure 3). Patency of the pLVB construct is confirmed with ICG imaging and those with arterial grafts are evaluated again in 15 min to confirm no back-bleeding due to the absence of valves.

### 2.3 Breast cancer-related lymphedema surveillance protocol

At our institution, BCRL surveillance is conducted through a structured, multidisciplinary program involving surgical oncologists, reconstructive microsurgeons with expertise in lymphatic surgery, and certified lymphedema therapists. All patients undergoing ALND were evaluated preoperatively to establish baseline measurements and subsequently followed at standardized postoperative intervals at 3, 6, 9, 12, 18, 24, and 36 months. Surveillance assessments included objective limb measurements using both circumferential measurements and perometry. Circumferential measurements were obtained at seven standardized anatomic landmarks of the bilateral upper extremities: middle finger, palmar crease, wrist, forearm, elbow, mid-arm, and axilla. Percent differences between the operative and contralateral limbs were calculated at each level. Limb volumetry was performed using a perometer system, which utilizes infrared light beams to generate limb volume. The percentage difference in volume between the affected and unaffected limbs was calculated for each assessment.

In our institution, lymphedema is defined as a greater than 15% difference in limb circumference measurements between the affected and contralateral extremity, a greater than 15% volume difference by perometry between the affected and contralateral extremity, or the need for therapeutic lymphatic reconstruction at any point after undergoing the pLVB procedure (11).

### 2.4 Statistical analysis

Statistical analyses were performed using R software (R Core Team, 2024). Descriptive statistics were used to summarize patient demographics, oncologic characteristics, operative variables, and outcomes. Normality of continuous variables was assessed using the Shapiro–Wilk test. Continuous variables were reported as mean ± standard deviation when normally distributed and as median (interquartile range) when non-normally distributed. Group comparisons were performed using Welch *t* tests for normally distributed continuous variables and Wilcoxon rank-sum tests for non-normally distributed continuous variables. Categorical variables were compared using Fisher exact tests. A value of *P* < .05 was considered statistically significant. Given the exploratory and feasibility-focused nature of this study, analyses were primarily descriptive, and no adjustments were made for multiple comparisons.

## 3 Results

### 3.1 Patient and oncologic characteristics

A total of 32 patients with a mean age of 52.3 (SD, 12.9) years and median BMI of 26.9 kg/m^2^ (IQR, 23.8–28.7) underwent pLVB with the use of a venous or arterial grafts at the time of ALND (Table 1) from 2019 to 2022. The mean of lymph nodes removed was 16.7 ± 6.9, with a median 1 (IQR, 0–5.25) positive lymph nodes. Most patients received neoadjuvant chemotherapy (81.0%), while neoadjuvant radiation was uncommon (9.4%). Adjuvant chemotherapy and radiation were administered in 75.0% and 90.6% of patients, respectively. The median follow-up duration was 15 months (IQR, 11–31).

**Table 1:** Demographic, oncologic, surgical, and outcome characteristics of patients who underwent pLVB with axillary-based interposition grafts.

Patient Characteristics			Oncologic Characteristics						Surgical Characteristics											
Patient ID	Age (years)	BMI (kg/m^2^)	Positive Nodes (No.)	Nodes Removed (No.)	Neoadjuvant Chemotherapy	Adjuvant Chemotherapy	Neoadjuvant Radiation	Adjuvant Radiation	Graft Type	Graft Donor Vessel	Graft Length (cm)	Graft Recipient Vessel	Lymphatic Vessels Identified (No.)	pLVB No.	Graft pLVB Lymphatic Diameter (mm)	Graft pLVB Anastomotic Technique	Additional Procedures	Operative Duration (min)	Follow-up Duration (months)	Lymphedema Development
**1**	34	22.3	0	15	Yes	No	No	Yes	Venous	Lateral thoracic vein	6	Axillary vein	6	3	0.5, 0.5, 0.5	Intussusception	L Mastectomy, L ALND	216	17	No
**2**	47	22.8	0	4	Yes	Yes	No	Yes	Arterial	Unspecified arterial branch	5	Axillary vein	11	2 graft, 9 non-graft	0.2–0.4	Intussusception	L ALND, L TE exchange, R port removal, R mastopexy	259	35	No
**3**	60	26.3	20	25	No	Yes	No	Yes	Arterial	Thoracodorsal artery	5	Axillary vein	7	1 graft, 6 non-graft	0.8	E-E	R Lumpectomy, R ALND	258	15	No
**4**	36	48.8	16	23	Yes	Yes	No	Yes	Venous	Serratus branch of thoracodorsal vein	3	Lateral thoracic vein	2	2	0.8, 0.6	Intussusception	R Mastectomy, R ALND	387	26	No
**5**	43	23.1	1	1	Yes	No	Yes	Yes	Venous	Thoracodorsal vein subcutaneous branch	4	Lateral thoracic vein	9	5 graft, 4 non-graft	0.3, 0.3, 0.5, 0.6, 0.6	Intussusception	B/l mastecomy, B/l TE placement	505	20	No
**6**	78	21.7	2	17	Yes	No	No	No	Venous	Lateral thoracic vein	4.5	Lateral thoracic vein	5	5	0.4, 0.5, 0.6, 0.6, 0.8	Intussusception	R Mastectomy, R ALND	220	4	No
**7**	30	22.9	1	17	Yes	Yes	No	Yes	Venous	Lateral thoracic vein	6	Axillary vein	3	1 graft, 2 non-graft	0.4	Intussusception	B/l mastectomy, L ALND, R SNLB	391	12	No
**8**	76	26.7	6	17	Yes	Yes	Yes	Yes	Arterial	Unspecified arterial branch	7	Axillary vein	10	3	0.3, 0.4, 0.5	Intussusception	L ALND	258	8	No
**9**	66	24.3	4	13	Yes	Yes	No	Yes	Arterial	Unspecified arterial branch	2	Axillary vein branch	5	2 graft, 3 non-graft	0.4, 0.5	Intussusception	L Mastectomy, L ANLD	305	15	No
**10**	49	32.9	11	13	Yes	Yes	Yes	No	Venous	Axillary vein branch	4	Axillary vein branch	2	2	0.3, 0.5	Intussusception, E-E	R Mastectomy, B/l ALND	326	16	No
**11**	71	27	3	13	No	Yes	No	Yes	Venous	Lateral thoracic vein	7	Lateral thoracic vein	2	2	0.3, 0,3	Intussusception	R Lumpectomy, R ALND	263	31	No
**12**	71	30.1	6	16	No	Yes	No	Yes	Arterial	Axillary artery branch	4	Axillary vein branch	2	1 graft, 1 non-graft	0.4	Intussusception	L Mastectomy, L ALND	327	2	No
**13**	59	25.3	1	21	Yes	Yes	No	Yes	Arterial	Lateral thoracic artery branch	—	Axillary vein branch	3	3	0.8, 0.9, 1	Intussusception	B/l mastectomy, L ALND	353	6	No
**14**	66	25.3	1	24	Yes	Yes	No	Yes	Arterial + venous	Unspecified arterial and venous branches	4 (vein), 5 (artery)	Arterial to venous graft, venous graft to thoracodorsal vein branch	5	5	0.2, 0.3, 0.5, 0.7, 0.7	Intussusception	R ALND	218	11	Yes
**15**	49	23.6	29	29	Yes	Yes	No	Yes	Venous + venous	Unspecified venous branch + intercostal vein branch	7 (unspecified), unspecified (intercoastal)	Venous graft to venous graft, venous graft to axillary vein	3	3	0.3, 0.5, 0.6	Intussusception	R Mastectomy, R ALND	348	46	Yes
**16**	61	27,6	1	11	No	Yes	No	Yes	Venous	Lateral thoracic vein	6	Axillary vein branch	6	3 graft, 3 non-graft	—	Intussusception	R ALND	214	12	No
**17**	58	27.2	0	27	Yes	No	No	Yes	Venous	Lateral thoracic vein	5	Thoracodorsal vein	4	4	0.2, 0.2, 0.45, 0.6	Intussusception	L Mastectomy, L ALND	349	6	Yes
**18**	56	30.7	1	15	Yes	Yes	No	Yes	Venous	Unspecified venous branch	6	Axillary vein branch	6	6	0.4, 0.4, 0.4, 0.5, 0.6, 0.6	Intussusception	R Mastectomy, R ALND	260	14	Yes
**19**	46	28.6	12	14	Yes	Yes	No	Yes	Venous	Lateral thoracic vein	6	Axillary vein	5	5	—	x3 E-E, x2 Intussusception	L Mastectomy, L ALND, L TE placement	326	11	No
**20**	40	22.9	4	23	Yes	Yes	No	Yes	Venous	Lateral thoracic vein	7	Thoracodorsal vein branch	5	5	0.3–0.4	Intussusception	R Mastectomy, R ALND	350	11	Yes
**21**	38	42.6	0	3	Yes	Yes	No	Yes	Venous	Unspecified venous branch	3	Thoracodorsal vein	4	4	0.7,0.7,0.7,0.7	Intussusception	B/l Mastectomy, L ALND, R SNLB	392	35	No
**22**	44	23.9	2	16	Yes	Yes	No	Yes	Arterial	Axillary artery branch	5	Axillary vein	2	2	0.4, 0.6	Intussusception	B/l Mastectomy, R ALND	336	12	No
**23**	58	27.2	0	13	Yes	Yes	No	Yes	Arterial	Thoracodorsal artery branch	—	Thoracodorsal vein	3	1 graft, 2 non-graft	—	Intussusception	R Mastectomy, R ALND, Excision of scalp & labial lesions	274	6	No
**24**	67	24.3	1	30	No	No	No	No	Venous	Unspecified venous branch	6	Axillary vein	3	3	—	Intussusception	R Mastectomy, R ALND, R breast DIEP flap reconstruction	657	19	No
**25**	36	20.8	0	10	Yes	Yes	No	Yes	Venous + venous	2 unspecifiied venous branches	—	Not specified	1	1	—	Intussusception	L Mastectomy, L ALND, L Chest wall TMR	377	31	No
**26**	55	27.1	6	25	Yes	No	No	Yes	Venous	Unspecified venous branch	2	Not specified	1	1	0.4	Intussusception	L Mastectomy, L ALND	274	44	No
**27**	39	26.6	0	19	Yes	Yes	No	Yes	Venous	Serratus branch of thoracodorsal vein	5	Lateral thoracic vein	4	4	0.4, 0.4,0.5, 0.5	Intussusception	R Mastectomy, R ALND, R breast msTRAM flap reconstruction	516	43	No
**28**	51	28.7	3	16	No	Yes	No	Yes	Venous	Lateral thoracic vein branch	3	Thoracodorsal vein	1	1	0.7	E-E	L Mastectomy, L ALND	404	18	No
**29**	56	33.4	0	18	Yes	Yes	No	Yes	Venous	Thoracodorsal vein branch	6	Serratus branch of thoracodorsal vein	2	1	0.4, 0.4	Intussusception	B/l Mastectomy, R ALND	265	12	No
**30**	46	38.8	0	18	Yes	No	No	Yes	Venous	Thoracodorsal vein branch	3	Axillary vein branch	1	1	0.7	Intussusception	L lumpectomy, L ALND, R Reduction mammaplasty	288	1	No
**31**	38	27	5	16	Yes	Yes	No	Yes	Venous	Lateral thoracic vein	5	Axillary vein branch	2	2	0.5, 0.7	Intussusception	B/l Mastectomy, L ALND, B/l TE placement,	424	40	Yes
**32**	51	28.7	0	13	Yes	No	No	Yes	Venous	Lateral thoracic vein	6	Axillary vein branch	6	5	0.3–0.6	Intussusception	R Mastectomy, R ALND	332	54	No

---: not reported/specified; pLVB, prophylactic lymphovenous bypass; E-E, end-to-end anastomosis; L, left; R, right; B/l, bilateral; ALND, axillary lymph node dissection; TE, tissue expander; DIEP, deep inferior epigastric; msTRAM, muscle-sparing transverse rectus abdominis.

ᵃ Graft: pLVBs anastamosed to the graft vessel; non-graft: pLVBs anastamosed to branch(es) of the graft recipient vessel.

### 3.2 Surgical characteristics

Across all cases, 121 transected lymphatic vessels were identified intraoperatively, of which 119 (98.3%) were successfully anastomosed (Table 2). Of the 119 anastomoses, the majority of lymphatic reconstructions (74.8%) required interposition grafts due to insufficient length or unfavorable characteristics of remaining axillary recipient veins following ALND. A total of 35 grafts were utilized, including 26 venous grafts (74.3%) and 9 arterial grafts (25.7%) with a median graft length of 5 cm (IQR, 4–6) (Table 3). Two patients required harvest of two venous grafts, and one patient required both venous and arterial grafts, which were sequentially anastomosed to extend reach to suitable recipient vessels.

**Table 2:** Summary of patient characteristics.

Variable	Value
**Patient Characteristics**	
Total patients, N (%)	32 (100)
Age, years, mean ± SD	52.3 ± 12.9
BMI, kg/m^2^, median (IQR)	26.9 (23.8–28.7)
**Oncologic Characteristics**	
Positive lymph nodes, median (IQR)	1 (0–5.25)
Lymph nodes removed, mean ± SD	16.7 ± 6.9
Neoadjuvant chemotherapy, N (%)	26 (81.3)
Adjuvant chemotherapy, N (%)	24 (75)
Neoadjuvant radiation, N (%)	3 (9.4)
Adjuvant radiation, N (%)	29 (90.6)
**Surgical Characteristics**	
Total lymphatic vessels identified, N (%)	121 (100)
Lymphatic vessels identified, median (IQR)	3.5 (2–5.25)
Total pLVB, N (%)	119 (100)
Total pLVB, median (IQR)	3 (2–5)
Graft pLVB, N (% total pLVB)	89 (74.8)
Graft pLVB, median (IQR)	2.5 (1–4)
Non-graft pLVB, N (% total pLVB)	30 (25.2)
Non-graft pLVB, median (IQR)	3 (2–4.5)
Operative duration, minutes, median (IQR)	326.5 (262.3–379.5)
**Follow-up duration, months, median (IQR)**	15 (11–31)
**Lymphedema Development, N (%)**	6 (18.8)

**Table 3:** Characteristics of interpositional grafts.

Graft Characteristics	Value
**Total, N (%)**	35 (100)
**Length, cm, median (IQR)**	5 (4–6)
**Venous, N (%)**	26 (74.3)
Lateral thoracic, N (% venous)	11 (42.3)
Thoracodorsal, N (% venous)	5 (19.2)
Axillary branch, N (% venous)	1 (3.8)
Intercostal branch, N (% venous)	1 (3.8)
Unspecified branch, N (% venous)	8 (30.8)
**Arterial, N (%)**	9 (25.7)
Thoracodorsal, N (% arterial)	2 (22.2)
Axillary, N (% arterial)	2 (22.2)
Lateral thoracic, N (% arterial)	1 (11.1)
Unspecified branch, N (% arterial)	4 (44.5)

Venous graft donor vessels most commonly originated from the lateral thoracic vein (42.3%), followed by the thoracodorsal vein (19.2%), axillary vein (3.8%), intercostal veins (3.8%), and unspecified venous branches (30.8%). Arterial grafts were harvested from the thoracodorsal artery (22.2%), axillary artery (22.2%), lateral thoracic artery (11.1%), and unspecified arterial branches (44.5%). The most frequently utilized recipient vessel was the axillary vein (56.3%), followed by the thoracodorsal vein (21.9%), lateral thoracic vein (15.6%), and other unspecified venous targets (6.2%).

A median of 3 pLVBs (IQR, 2–5) were performed per patient, with a median of 2.5 (IQR, 1–4) lymphatic vessels anastomosed to grafts. Among 66 pLVBs anastomosed to grafts in which lymphatic diameter was reported, the median diameter was 0.5 mm (IQR, 0.4–0.6). All graft pLVBs were anastomosed with intussusception (93.3%) or end-to-end (6.7%) techniques, and patency was confirmed in all cases by visualization of ICG transit across the anastomosis. The median total operative duration was 326.5 min (IQR 262.3–379.5) and is reflective of all procedures, including reconstructive procedures performed in adjunct to graft harvest and lymphatic reconstruction in 28.1% of patients.

### 3.3 Outcomes

The overall incidence of BCRL was 18.8% (*N* = 6) (Table 4). Perometer measurements were available for 5 of 6 patients who developed lymphedema. Although the affected limb demonstrated a median 0.9% (IQR, −0.9% to 4.3%) greater volume compared with the contralateral limb in these patients, all 5 patients exhibited clinically significant lymphedema requiring additional lymphovenous bypass procedures. For the remaining patient without perometer data, circumferential measurements demonstrated increased girth at the middle finger (+49.1%), palmar crease (+4.7%), wrist (+7.1%), forearm (+15.8%), elbow (+7.2%), and upper arm (+7.9%), with a slight decrease at the axilla (−1.8%) compared with the unaffected limb.

**Table 4:** Comparative analysis of patients with and without lymphedema.

Variable	No lymphedema (*N* = 26)	Lymphedema (*N* = 6)	*P*
**Patient Characteristics**			
Age, years, mean ± SD	52.6 ± 13.5	51.2 ± 10.9	0.786
BMI, kg/m^2^, median (IQR)	26.9 (24.0–28.7)	26.1 (24.0–27.1)	0.717
**Oncologic Characteristics**			
Positive lymph nodes, median (IQR)	1 (0–6)	2 (1–5)	0.538
Lymph nodes removed, mean ± SD	**15.4** **±** **6.6**	**22.3** **±** **5.7**	**0**.**031**
Neoadjuvant chemotherapy, N (%)	20 (76.9)	6 (100.0)	1
Adjuvant chemotherapy, N (%)	19 (73.1)	5 (83.3)	1
Neoadjuvant radiation, N (%)	3 (11.5)	0 (0.0)	1
Adjuvant radiation, N (%)	23 (88.5)	6 (100.0)	1
**Surgical Characteristics**			
Lymphatic vessels identified, median (IQR)	3 (2–6)	4.5 (3–5)	0.591
Total pLVB, median (IQR)	3 (2–5)	4.5 (3–5)	0.292
Graft pLVB, median (IQR)	**2** (**1–3)**	**4.5** (**3–5)**	**0**.**023**
Non-graft pLVB, median (IQR)	3 (2–5)	NA	NA
Graft pLVB diameter, mm, median (IQR)	0.5 (0.4–0.6)	0.5 (0.4–0.6)	0.8737
Operative duration, minutes, median (IQR)	326 (264–385)	348 (282–350)	0.866
**Graft Characteristics**			
Venous, N (%)	17 (65.4)	4 (66.6)	0.0785
Arterial, N (%)	8 (30.8)	0 (0)	0.0785
Venous + venous, N (%)	1 (3.8)	1 (16.7)	0.0785
Arterial + venous, N (%)	0 (0)	1 (16.7)	0.0785
Length, cm, median (IQR)	5 (3–6)	5.5 (5–7)	0.162
**Follow-up duration, months, median (IQR)**	15.5 (18.5)	12.5 (22.5)	0.942

Bolded values indicate significant findings (*P* < .05).

When comparing patients with and without lymphedema, those who developed BCRL had a significantly higher mean number of lymph nodes removed (22.3 vs. 15.4; *P* < .05) and a higher median number of graft-based pLVBs (4.5 vs. 2; *P* < .05). No statistically significant differences were observed with respect to age, BMI, number of positive lymph nodes, neoadjuvant or adjuvant systemic therapy, total number of pLVBs, lymphatic vessel diameter, graft characteristics, operative duration, or follow-up duration.

## 4 Discussion

Despite the increasing role of prophylactic lymphatic reconstruction to prevent the development of BCRL, this procedure remains technically demanding given the relative lack of adequate recipient veins secondary to inadequate length, vessel mismatch, and venous pressure difference (10, 14–17). While prior studies have demonstrated that interposition grafts lead to a decrease in the pLVB procedure abortion rate, non-axillary vein grafts require a second operative site (10, 14). In contrast, our institutional approach utilizes vascular grafts harvested entirely within the axillary dissection field, where these vessels are frequently already transected or exposed during ALND. This strategy avoids secondary donor sites while providing access to a wide range of graft options in terms of length, caliber, and branching patterns within the operative field.

In our study, we reviewed the outcomes of 32 patients who underwent axillary vascular grafts for pLVB from 2019 to 2022. To the best of our knowledge, this is the largest report of using axillary vascular grafts for preventive lymphatic surgery for breast cancer-related lymphedema. Our results indicate that interpositional grafts from the axilla lead to successful preventive lymphatic reconstruction while minimizing the abort rate due to the lack of recipient veins. Of the 121 transected lymphatic vessels, vascular grafts were utilized in 74.8% of the lymphatic reconstructions, leading to a median of 3 pLVBs being performed per patient, with a median 2.5 anastomosed to grafts. This finding supports that lymphatic-to-vein anastomosis without a graft would have substantially limited the extent of reconstruction achievable in many patients following ALND. In a review of 39 studies, Hinson et al. reported an average of 2.11 anastomoses per patient, which our cohort was able to achieve through the use of interposition grafts (median 3 pLVBs) (16). In addition, our cohort includes eight patients in which the use of interposition grafts resulted in a 53% increase in the number of lymphatic anastomoses created for a total of 46 pLVBs, which highlights the importance and advantages of using grafts. While the exact threshold of anastomoses required to effectively prevent BCRL remains undefined, a recent systematic review and meta-analysis reports that the number of anastomoses performed across ILR studies ranged from 1 to 4, placing our cohort's median of 3 pLVBs per patient within range of what has been reported in the literature (17). Furthermore, our results demonstrated that those with a higher number of pLVBs had a lower incidence of lymphedema, which is consistent with prior work suggesting that a greater number of functional anastomoses confers a lower BCRL risk (11). Collectively, our findings indicate that axillary interposition grafting expands achievable lymphatic reconstruction in an otherwise vein-depleted axilla.

The second aim of our study was to investigate the efficacy of using interpositional grafts to prevent the development of BCRL. In our study, the overall incidence of BCRL in our cohort was 18.8% (*N* = 6), which is within the range of rates reported in studies of ILR and pLVB (3–31.1%) (18, 19). It should be noted that our cohort represents a selected subgroup of patients with vein-depleted axillae, likely reflecting a higher-risk population with greater lymphatic disruption. Patients who developed BCRL had a significantly higher mean number of lymph nodes removed (22.3 vs. 15.4; *P* < .05). This finding is in agreement with the established association between more extensive axillary surgery and greater lymph node removal as risk factors for BCRL development (1, 2, 20). These patients also had a greater median number of pLVBs performed using interpositional grafts (4.5 vs. 2; *P* < .05), indicating that they experienced greater lymphatic disruption and had unfavorable axillary anatomy. Moreover, there were higher rates of neoadjuvant chemotherapy (100% vs. 76.9%), adjuvant radiation (100% vs. 88.5%), and adjuvant chemotherapy (83.3% vs. 76.1%) among this group, likely indicating more aggressive disease. Although these differences did not reach statistical significance in this small cohort, there is a known link between increasingly aggressive multimodal therapies and lymphedema risk (21–23). Taken together, these findings highlight the need for improved risk stratification and patient selection criteria to better identify those who derive the greatest benefit from preventive lymphatic reconstruction.

Additionally, we evaluated the versatility and technical feasibility of the axillary grafts. 74.3% of grafts were venous, with the lateral thoracic vein being the most commonly harvested (42.3%). Arterial grafts were used selectively when venous options were limited or unsuitable. In our experience, the lateral thoracic vessels are ideal vascular grafts due to their small caliber (1.2–1.5 mm) (24), multiple branching patterns (0–3 branches, mean: 1.8), long length (3–7 cm, mean: 5.4), and proximity to the lymphatic node basin (25). Due to its branching pattern, it's useful as a graft near the lymphatic node basin, where various types of functional lymphatic vessels are severed. The second graft vessels of choice are the perforating chest wall vessels at the lateral, superior border of pectoralis muscle, which are easily accessible and available during the ALND. While thoracodorsal vessels are readily accessible, they were used selectively in patients with no alternative venous options were available in order to preserve thoracodorsal pedicle for latissimus dorsi-based breast reconstruction. For all grafts, we monitored for the patency and potential back-bleeding of the anastomosis in length to ensure the success of the pLVB as the branches of the axillary vein have been found to have less valves than that of the lower extremity (26, 27).

Lastly, we found that use of axillary grafts offers practical advantages in operative workflow. First, this approach eliminates the need for additional incisions and donor-site management while decreasing our surgery abortion rate to 0% and increase trainee participation by division of the work. In this approach, the primary surgeon will investigate the operative field in a systematic manner to identify, harvest, and prepare the graft while including its tributary branches for an improved vessel match to lymphatic vessels while the assisting surgeon, typically a resident or microsurgery fellow, will locate the functional lymphatic vessels that have been transected. This operative advantage is demonstrated in our median total operative duration of 326.5 min, which includes time for oncologic resection, axillary lymph node dissection, lymphatic reconstruction and breast reconstruction if indicated. While we did not record the exact operative time required for lymphatic reconstruction specifically, we anticipate that our operative efficiency will continue to improve as we reduce the time spent on searching for suitable neighboring veins and performing anastomosis using unsuitable recipient veins by utilizing axillary interpositional grafts. This phenomenon was observed by Shaffer *et al*. in performing lymphatic preventive surgery, and we believe that a similar learning-curve effect may reasonably be expected for procedures requiring interposition graft harvest (28).

### 4.1 Limitations

Our study was not without limitations. The retrospective, single-institution design and relatively small sample size introduce inherent selection bias and limit the ability to establish relationships and draw definitive causal inferences regarding lymphedema development. Although patients were retrospectively identified for inclusion, prospective recruitment and the use of a standardized postoperative institutional protocol enhanced the accuracy and consistency of outcomes data. Additionally, our cohort represents a selected population in whom graft-based pLVB was required, reflecting more complex axillary anatomy than typical pLVB candidates, which limits generalizability to patients undergoing standard pLVB. With regards to technique, we were not able to conduct meaningful analyses due to the small sample size, and future studies with larger cohorts are needed. Therefore, larger-scale prospective studies are needed to validate the technical feasibility and reproducibility of axillary-based interposition grafts use, and its long-term impact on lymphedema outcomes.

## 5 Conclusion

In this study, we report our experience with utilizing axillary vascular grafts to perform 119 pLVBs immediately following ALND in 32 patients. We found that the use of interposition grafts led to a 0% abort rate and increased the number of successful pLVBS in an anatomically complex and vein-depleted axilla. By leveraging venous and arterial conduits already present within the operative field, this approach allows a practical and versatile strategy to overcome one of the most common technical barriers to pLVB and to maximize the extent of ILR in patients at elevated risk for lymphedema. We hope that this technique will improve the armamentarium of surgeons who perform immediate lymphatic reconstruction and are met with technical challenges.

## Data Availability

The original contributions presented in the study are included in the article/Supplementary Material, further inquiries can be directed to the corresponding author.
